# Quality of life in pediatric patients on a paracorporeal ventricular assist device with a novel mobile driving system

**DOI:** 10.1016/j.jhlto.2024.100125

**Published:** 2024-07-17

**Authors:** Oliver Miera, Eugen Sandica, Nikolaus A. Haas, Martin Schweiger, Brigitte Stiller, Rainer Kozlik-Feldmann, Maria-Helena Perez, Ina Michel-Behnke, Katharina R.L. Schmitt, Stephan Schubert, Daniel Zimpfer

**Affiliations:** a Department of Congenital Heart Disease - Pediatric Cardiology, Deutsches Herzzentrum der Charité, Berlin, Germany; b Department of Pediatric Heart Surgery and Surgery for Congenital Heart Defects, Heart, and Diabetes Center NRW, Bad Oeynhausen, Germany; c Department of Pediatric Cardiology and Pediatric Intensive Care, Ludwig Maximilian University of Munich, Munich, Germany; d Department of Congenital Cardiovascular Surgery, Pediatric Heart Center, University Children's Hospital Zurich, Switzerland; e Department of Congenital Heart Defects and Pediatric Cardiology, University Heart Center Freiburg-Bad Krozingen, Freiburg, Germany; f Clinic for Children's Heart Medicine and Adults with Congenital Heart Disease, University Heart & Vascular Center, Hamburg, Germany; g Women-Mother-Child Department, University Hospital of Lausanne, Lausanne, Switzerland; h Department of Pediatric Cardiology, Medical University of Vienna, Wien, Austria; i Department of Pediatric Cardiology and Congenital Heart Defects, Heart, and Diabetes Center NRW, Bad Oeynhausen, Germany; j Department of Heart Surgery, Medical University of Vienna, Wien, Austria; k Division of Cardiac Surgery, University Heart Center, Graz, Austria

**Keywords:** ventricular assist device, pediatric, driving unit, mobility, quality of life

## Abstract

**Background:**

EXCOR ventricular assist device (VAD) is the gold standard circulatory support for children with end-stage heart failure. Until recently, the only available driving unit was the stationary Ikus. This study (NCT04634708) investigates the impact of the novel mobile EXCOR Active driving unit on patients’ mobility and the quality of life (QoL) of their families.

**Methods:**

Children on EXCOR VAD support with the Ikus who were mobilizable outside the hospital room were eligible for the prospective study. Patients remained on Ikus for 7 days, then switched to Excor Active, and were observed for another 45 days. The end-points were the rates of clinically relevant device exchanges (i.e., exchanges that could raise safety concerns for the patient) and successful patient outcomes, respectively. QoL of patients’ families was recorded through 2 validated questionnaires: the Pediatric Quality of Life Inventory Family Impact Module and Depression, Anxiety and Stress Scale 21. Patients’ mobility and activity levels were monitored through diary entries.

**Results:**

A total of 24 patients were enrolled of which 23 patients completed the study. No clinically relevant device exchanges occurred and there was a successful outcome in 22/23 (95.7%) patients. Changing from the Ikus to the EXCOR Active improved QoL as seen in family impact total score (from 53.8 ± 19.8-66.5 ± 20.8, *p* = 0.005) and mental health (depression: moderate to mild, stress moderate to normal, both *p* < 0.05; anxiety: mild to normal, n.s.). Activity levels raised in both, mean time (from 100-300 min/day, *p* = 0.011) and distance (from 30-1,110 m/day, *p* = 0.002).

**Conclusions:**

The EXCOR Active driving unit is safe and performs equivalent to Ikus. This study indicates considerable improvement in patients’ mobility. In addition, data suggest that families’ QoL is positively affected by means of the novel driving unit.

## Background

Heart failure (HF) is a significant cause of morbidity and mortality in the pediatric population,^1^ which affects 12,000 to 35,000 children below age of 19 years in the United States.^2^, ^3^ The estimated incidence in Europe lies between 0.87 and 3 per 100,000 children.^4^ Additionally, pediatric HF is at the same time a health care and a social issue due to the associated cost of care for the society^1^ and psychological burden to the families and parental caregivers.^5^ While the medical therapy has improved the outcomes, the only final treatment option for children with refractory end-stage HF remains heart transplantation (HTx).^1^ However, due to the limited number of donors and suitable organs, wait time on HTx listing is often lengthy and associated with high mortality rates.^6^ As a consequence, the use of mechanical circulatory support (MCS) in children has considerably increased as a bridge to HTx, allowing for patient stabilization and pre-HTx conditioning while on the waitlist.^7^

Nowadays, the EXCOR Ventricular Assist Device (VAD, Berlin Heart, Germany) is the gold standard in durable MCS for children. The EXCOR VAD is a paracorporeal, pulsatile device that can support pediatric patients of all ages. For small children and infants, it is the only approved cardiac assist device available worldwide.^8^ The EXCOR VAD therapy is established for decades in the pediatric population showing significant beneficial results in terms of patient survival and morbidity reduction.^9^, ^10^, ^11^ As being pneumatically driven, it requires a connection to an external driving unit. The Ikus Stationary Driving Unit (Berlin Heart) fulfills this purpose, but its size and limited battery life restrict patient mobility. Moreover, restrictions may arise from safety concerns, that is, anxiety about the device integrity or dislodgement during mobilization.^12^ Therefore, children on paracorporeal VAD support exhibit significant impairment in mobility, exercise, and playing possibilities due to the presence of the operating console (driving unit).^13^, ^14^ VAD patients may be disturbed by the hindered mobility and by the inability to participate in playing activities,^14^ which qualifies as a large quality of life (QoL) deficit compared with outpatient management of severe heart disease or post heart transplant patients.^15^ The recent advances in the VAD field have strongly pushed toward the improvement of patient’s QoL as one of the pivotal aspects of a VAD therapy. This request was translated for the EXCOR VAD as the need—expressed by patients, families, and hospital staff—to improve patient mobility and patient’s freedom to perform the everyday activities.^16^

The EXCOR Active driving system (Berlin Heart) is a novel, portable driving unit specifically designed for mobilization. It is smaller, lighter, has a longer battery life than the Ikus (Table 1), and can be used with additional mobilization devices, such as a caddy or a baby buggy, according to patient’s age. The performance and safety of the EXCOR Active as well as its impact on patient mobility and family QoL were investigated in this clinical trial entitled “The Use of the EXCOR Active Driving Unit for Mobilization of Pediatric Patients with Ventricular Assist Device Support” (“E-Motion” Study, NCT04634708).

**Table 1:** Overview of Different Parameters of the Two Driving Units Ikus and EXCOR Active

Parameter	Ikus driving unit	EXCOR Active driving unit
Dimension (W × H × D)	46 × 95 × 73 cm	33 × 40 × 22 cm
Weight	90 kg	15 kg
Off-mains operating time (BVAD mode)	Max 0.5 h	Min 4:30 h (adult) Min 6:30 h (children)

Abbreviations: BVAD, biventricular assist device.

For further technical details, please see https://www.berlinheart.de/fileadmin/user_upload/Berlin_Heart/Dokumente/Downloads/Downloads_IFU/EXCOR_Active/clinic/1015002x03_A04_EXCOR_Active_GA_Klinik_en.pdf.

## Patients and methods

This study is an international, multicenter, prospective, open-label, nonrandomized, single-arm, observational study in the pediatric population. The study was conducted in full accordance with Good Clinical Practice principles, laid down in the ISO 14155, at 8 sites in 3 countries. The study commenced in November 2020 after approval by the responsible independent ethics committees.

Patients were eligible for inclusion if they were on support with the EXCOR VAD and the Ikus, were younger than 18 years of age, and were able to get mobilized according to hospital protocol. Mandatory informed consent was obtained before enrollment from a legal guardian (parents or carers) and from the patient by means of an age-specific form. Exclusion criteria were any contraindications to being supported by the EXCOR Active as specified in the manufacturer’s instructions, lack of consent from the patient or legal guardian, or being a female of childbearing age who was either pregnant or not on contraceptives or surgically sterile.

Data of the enrolled patients were collected at 3 timepoints. The subjects were enrolled in the study (visit 1) after they were able to get mobilized. Patients remained on the Ikus for the next 7 days, were then switched to the EXCOR Active (visit 2), and then followed up for another 45 days of support (until visit 3).

The primary end-point of the study was the rate of clinically relevant driving unit exchanges. Owing to the novelty of the device, exchanges originating from nonrelevant issues (e.g., software bugs, cosmetic reasons, increased safety precautions taken by the investigators) were expected. Thus, each device exchange was assessed by an expert group with regards to its clinical relevance. Device exchanges were assessed as clinically relevant if the issue raised safety concerns for the patients. In particular, the rate of the EXCOR Active exchanges was compared to the rate of clinically relevant Ikus exchanges. The secondary end-point consisted of the rate of successful outcomes, that is, HTx, recovery and weaning from the device, and alive on device support at the end of the observation period. The safety end-point included the assessment of all adverse events (AE) and serious AE rates, including the evaluation of whether the relationship with the EXCOR Active, the EXCOR VAD, or the procedure of switching the driving units at visit 2 was at least possible. The rate of EXCOR Active deficiencies was also evaluated. The Medical Dictionary for Regulatory Activities (MedDRA) terminology was adopted to classify each AE. Additional end-points analyzed the general well-being of the patients and their families (by means of QoL assessment) and the patients’ mobility. The QoL was assessed through 2 validated questionnaires (i.e., the Pediatric Quality of Life Inventory [PedsQL] Family Impact Module^17^; the Depression, Anxiety and Stress Scale 21 [DASS 21]^18^) that were filled out by the parents. The 8 dimensions of the PedsQL Family Impact Module are reported individually and summarized with 3 total scores (Total Score, Parent health-related quality of life [HRQL], Summary Score, and Family Functioning Summary Score). The DASS 21 is a questionnaire that provides separate scores on depression, anxiety, and stress. The mobility of the patients was evaluated through self-reported diaries updated by the patient and/or their parents or caregivers twice a week. The records included the number of performed activities, the time spent on each activity, distance covered from the ward, and independence from the company and support of a health care professional (HCP).

The study analyzed the intraindividual changes by means of common descriptive statistical methods (i.e., Wilcoxon Signed Rank Test). Continuous variables were summarized as means, standard deviations, medians, and ranges according to the data distribution. Categorical variables were summarized as frequencies and percentages. End-point–related outcomes were reported with 95% confidence intervals (CIs). The study does not allow for confirmative conclusions. Competing outcome analysis was performed to show the proportion of transplanted, weaned from the device, on system, and deceased patients until study conclusion.

## Results

### Baseline information

A total of 24 patients were enrolled in this study. The median day of enrollment was 19.5 days after EXCOR VAD implantation (interquartile range [IQR], 13.5, 41.5). Patients were switched to EXCOR Active on day 29 (IQR 20, 57) and completed the study on day 72 (IQR 66, 110) postimplantation. Of the 24 enrolled patients, 23 patients completed the study, whereas 1 patient was discontinued due to an early HTx 1 day after study inclusion. As such, the latter patient was excluded from the end-point analyses except for the safety analysis. The patients ranged from newborns to adolescents with a median age of 1.66 (range: 0.0, 17.4) years (Table 2). Most subjects (54.2%) were aged between 1 and 24 months. Distribution by gender was comparable (male, 54.2%; female, 45.8%). The most common primary diagnosis was cardiomyopathy (79.2%). Before the EXCOR VAD implantation, 79.2% of the patients were on inotropic support and 54.2% received mechanical ventilation. At the time of implantation, 50.0% of the patients received the EXCOR as a left VAD, 45.8% as a biventricular VAD, and 4.2% as a right VAD.

**Table 2:** Baseline Characteristics of the E-Motion Study Population

Variable	*n* = 24
Age group, *n* (%)	
<1 month	1 (4.2%)
1-<24 months	13 (54.2%)
2-11 years	6 (25.0%)
12-18 years	4 (16.7%)
Male, *n* (%)	13 (54.2%)
BSA, m^2^, median (range)	
<1 month	0.24 (n.a., n.a.)
1-<24 months	0.43 (0.3, 0.6)
2-11 years	0.81 (0.6, 1.3)
12-18 years	1.25 (1.2, 1.4)
Primary cardiac diagnosis, *n* (%)	
Cardiomyopathy	19 (79.2%)
CHD	3 (12.4%)
Myocarditis	2 (8.3%)
Type of circulation, *n* (%)	
Univentricular physiology	3 (12.5%)
Biventricular physiology	21 (87.5%)
Medical history, *n* (%)	
Previous cardiac operations/cardiotomy	4 (16.7%)
Any impairment of heart valves	8 (33.3%)
Cerebrovascular events	2 (8.3%)
Infections	7 (29.2%)
Arrhythmias	7 (29.2%)
On inotropes	19 (79.2%)
On ECMO support	6 (25.0%)
On other types of MCS support	2 (8.3%)
On mechanical ventilation	13 (54.2%)
CPR within 7 days before implantation	7 (29.2%)
VAD configuration, *n* (%)	
BVAD	11 (45.8%)
LVAD	12 (50.0%)
RVAD	1 (4.2%)

Abbreviations: BSA, body surface area; BVAD, biventricular assist device; CHD, congenital heart disease; CPR, cardiopulmonary resuscitation; ECMO, extracorporeal membrane oxygenation; LVAD, left ventricular assist device; MCS, mechanical circulatory support; RVAD, right ventricular assist device.

### Primary end-point

There were no clinically relevant driving unit exchanges (Table S1). Thus, the primary end-point was not further evaluated. Of note, 1 patient had a nonclinically relevant exchange during the Ikus period (0.58 events/100 patient-days; 95% CI [0.01, 3.24]) while 4 patients had exchanges during the EXCOR Active period (0.46 events/100 patient-days (95% CI [0.13, 1.18]), resulting in a difference of 0.12 events/100 patient-days (95% CI [−1.01, 1.25]), which is not statistically significant (*p* = 0.836).

### Secondary end-point

**Figure 1:**
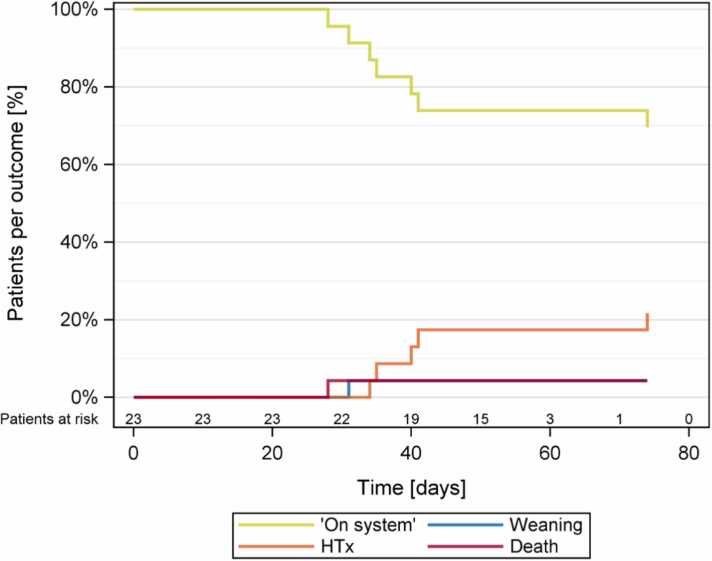
Competing outcomes are shown for patients on system, transplanted, weaned, and deceased during the observational period. HTx, heart transplantation.

Exactly 95.7% of the patients had a successful outcome: 69.6% were on EXCOR VAD support at the end of the follow-up, 21.7% had undergone HTx, and 4.3% were in the process of being weaned off the device (Figure 1). One patient died due to uncontrollable bleeding for causes not related to the EXCOR Active.

### Safety end-point

A total number of 27 AEs was reported and affected 13 patients (Table 3). The AE rate per patient day was 0.05 (95% CI [0.02, 0.10]) during the Ikus period and 0.02 (95% CI [0.01, 0.03]) while on EXCOR Active support, resulting in a statistically significant (*p* = 0.019) difference of 0.03 AEs/patient-days (95% CI [0.01; 0.06]).

**Table 3:** Overview of the 27 Adverse Events That Occurred During the Study Observational Period and Classified According to the Medical Dictionary for Regulatory Activities (MedDRA)

Adverse Event Summary	Visit 1 − Visit 2, *n* = 24		Visit 2 − Visit 3, *n* = 24	
MedDRA Term	Events	*n* (%)	Events	*n* (%)
Overall	9	7 (29.2)	18	10 (41.7)
Blood and lymphatic system disorders	0	0	0	0
Hemolysis	0	0	0	0
Gastrointestinal disorders	0	0	2	2 (8.3)
Diarrhea	0	0	1	1 (4.2)
Vomiting	0	0	1	1 (4.2)
General disorders and administration site conditions	0	0	3	3 (12.5)
Pyrexia	0	0	2	2 (8.3)
Administration site hematoma	0	0	1	1 (4.2)
Investigations	1	1 (4.2)	2	2 (8.3)
Hemoglobin decreased	1	1 (4.2)	0	0
Heart rate decreased	0	0	1	1 (4.2)
INR increased	0	0	1	1 (4.2)
Infections and infestations	2	2 (8.3)	3	3 (12.5)
Enterococcal infection	0	0	1	1 (4.2)
Erysipelas	0	0	1	1 (4.2)
*Escherichia* infection	0	0	1	1 (4.2)
Infection	1	1 (4.2)	0	0
Respiratory tract infection	1	1 (4.2)	0	0
Injury, poisoning, and procedural complication	1	1 (4.2)	0	0
Wound dehiscence	1	1 (4.2)	0	0
Nervous system disorders	1	1 (4.2)	2	1 (4.2)
Ischemic stroke	1	1 (4.2)	2	1 (4.2)
Product issues	4	3 (12.5)	2	2 (8.3)
Device deposit issue	4	3 (12.5)	2	2 (8.3)
Reproductive system and breast disorders	0	0	1	1 (4.2)
Balanoposthitis	0	0	1	1 (4.2)
Respiratory, thoracic, and mediastinal disorders	1	1 (4.2)	1	1 (4.2)
Epistaxis	1	1 (4.2)	1	1 (4.2)
Vascular disorders	0	0	1	1 (4.2)
Hemorrhage	0	0	1	1 (4.2)

Abbreviations: INR, international normalized ratio; MedDRA, Medical Dictionary for Regulatory Activities.

The numbers and percentages of patients with adverse events are summarized by system organ class and MedDRA preferred term. Visit 1 until visit 2 represents the Ikus period and visit 2 until visit 3 represents the EXCOR Active period.

Four AEs were classified as serious and included respiratory tract infection, ischemic stroke, deposit in the EXCOR VAD outflow cannula, and death due to uncontrollable bleeding at the truncus brachiocephalicus in a patient with congenital heart disease. Emergency revision surgery revealed lacerations in the truncus brachiocephalicus and the aortic arch that could not be closed due to fragility of the tissue. One AE was considered at least possibly related to the study procedure and occurred while on the Ikus support. The AE was caused by low hemoglobin levels and was treated with a transfusion. None of the serious AEs were assessed as related to the EXCOR Active. A total of 7 device deficiencies were reported for the EXCOR Active (Table S1).

### Assessment of the quality of life

**Figure 2:**
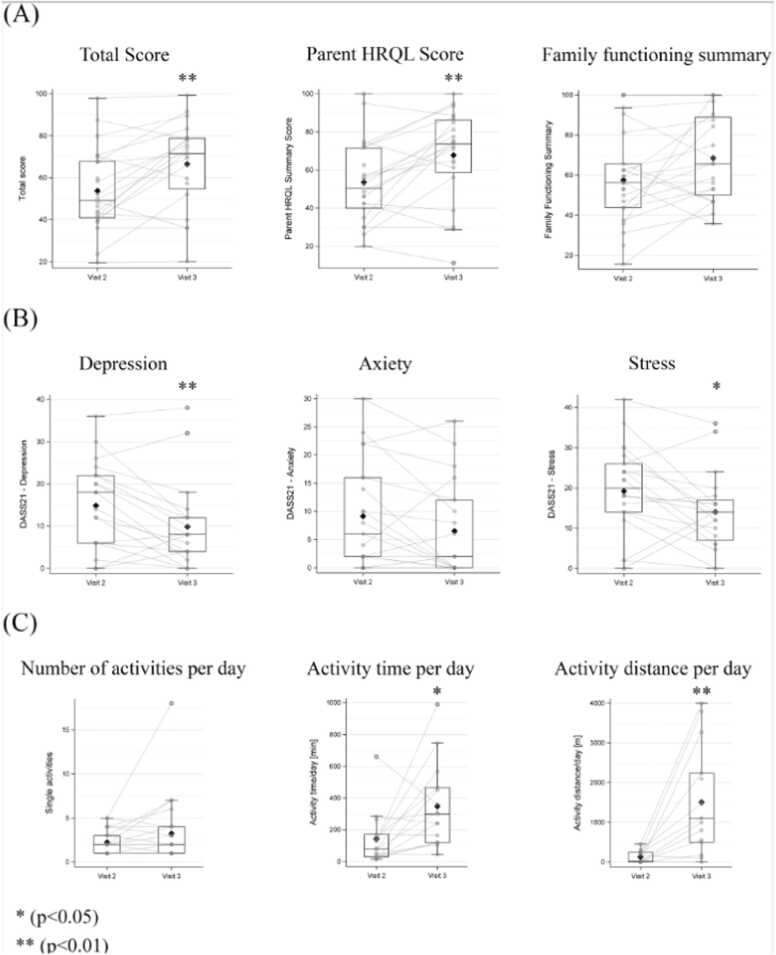
Scores from (A) PedsQL Family Impact Module, (B) DASS 21 questionnaires, and (C) diary data are illustrated separately for visit 2 (Ikus period) and visit 3 (EXCOR Active period). Shifts of individual patient scores are reported as light gray lines. Statistically significant changes in absolute values are calculated with the Wilcoxon Signed Rank Test: *p < 0.05, **p < 0.01. DASS 21, Depression, Anxiety and Stress Scale 21; HRQL, health-related quality of life; PedsQL, Pediatric Quality of Life Inventory.

An improvement in HRQL was observed after exchanging from the Ikus to the EXCOR Active (i.e., comparing visit 3 with visit 2), as measured by the PedsQL Family Impact Module. There were statistically significant differences between the 2 driving unit periods for the total score (*p* = 0.005) and the parent HRQL summary score (*p* = 0.004, Figure 2A). Additionally, scores of the emotional functioning, social functioning, cognitive functioning, worry, and daily activities dimensions show statistical significance in favor of EXCOR Active (Table 4).

**Table 4:** Scores of the Dimensions From the PedsQL Family Impact Module are Summarized as Mean ± SD at Visit 2 (Ikus Period) and Visit 3 (EXCOR Active Period)

Dimension		Visit 2	Visit 3	
	*n*	Mean ± SD	Mean ± SD	*p*-value
Physical functioning	19	53.07 ± 25.14	62.92 ± 27.30	0.073
Emotional functioning	19	47.44 ± 27.54	67.00 ± 28.35	<0.001^a^
Social functioning	19	50.28 ± 25.91	66.25 ± 27.39	0.016^a^
Cognitive functioning	19	63.18 ± 22.97	76.00 ± 23.15	0.021^a^
Communication	19	58.33 ± 23.43	71.25 ± 19.59	0.059
Worry	20	45.65 ± 22.33	55.25 ± 23.87	0.027^a^
Daily activities	17	33.88 ± 32.57	50.00 ± 27.95	0.022^a^
Family relationships	20	71.52 ± 21.92	77.06 ± 19.29	0.486
Parent HRQL summary score	19	53.60 ± 21.18	67.88 ± 24.26	0.004^a^
Family functioning score	20	57.53 ± 23.03	68.61 ± 21.67	0.142
Total score	19	53.82 ± 19.76	66.46 ± 20.77	0.005^a^

Abbreviations: SD, standard deviation; HRQL, health-related quality of life.

The number of available data for the change in dimensions is reported in the “*n*” column.

a *p*-values that are significant (<0.05) according to the Wilcoxon Signed Rank Test.

An overall improvement in changing from the Ikus to the EXCOR Active was also observed in patients’/caregivers’ depression, anxiety, and stress levels, as measured by the DASS 21 questionnaire (Figure 2B). Conventional severity labels (normal, moderate, severe, extremely severe) were based on the recommended cut-off scores.^17^ Depression levels experienced by patients/caregivers decreased from extremely severe (score: 14.86 ± 10.36) during the Ikus period to moderate (score: 9.89 ± 10.16, *p* < 0.05) during the EXCOR Active period. Anxiety levels decreased from severe/extremely severe (score: 9.14 ± 9.31) during the Ikus period to moderate (score: 6.53 ± 8.45, n.s.) during the EXCOR Active period. Stress levels also decreased, going from mild/moderate (score: 19.18 ± 11.32) during the Ikus period to normal (score: 14.03 ± 9.50, *p* < 0.05) during the EXCOR Active period.

**Figure 3:**
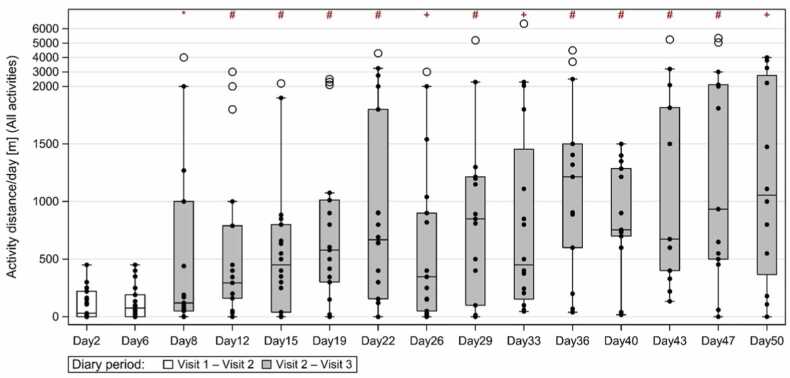
Activity distances [m] per diary day are displayed from visit 1 to visit 3. White boxes represent the period between visit 1 and visit 2 (Ikus period), gray boxes represent the period between visit 2 and visit 3 (EXCOR Active period). The plots show the median (middle line), quartiles (upper and lower boundaries of the box), range (whiskers), and outliers (circles). The y axis of the graph is manually scaled with values above 2,000 m compressed to 1/8 with respect to 0 to 2,000 m. Significance rating indicators in graph refer to *: *p* < 0.05, +: *p* < 0.01, #: *p* < 0.001.

While the number of overall activities remained unchanged, there were significant increases in the time spent doing daily activities and the distances covered on changing from the Ikus to the EXCOR Active (Table 5, Figure 2C). The median activity time per day overall increased from 100 (range: 15, 660) minutes to 300 (range: 45, 990) minutes (*p* = 0.011). The activity distance per day overall increased from 30 (range: 0, 450) m to 1,110 (range: 0, 4,000) m (*p* = 0.002). A shift in activity distance after the switch to EXCOR Active could be observed already in the first diary entry after the switch (Figure 3). These changes were driven by activities done without HCP company—differences in times and distances of activities done with HCP company were not statistically different between the 2 periods.

**Table 5:** Scores From the Diaries (Activity Sheets) are Summarized as Median (Range) at Visit 2 (Ikus Period) (i.e., First Diary Entry) and at Visit 3 (EXCOR Active period) (i.e., Last Diary Entry)

Activity	Visit 2		Visit 3		
	*n*	Median (range)	*n*	Median (range)	*p*-value
All activities					
No. of single activities	23	2 (1, 8)	23	2 (1, 18)	0.663
Activity time/day, minutes	21	100 (15, 660)	15	300 (45, 990)	0.011^a^
Activity distance/day, m	19	30 (0, 450)	12	1110 (0, 4,000)	0.002^a^
HCP company					
No. of single activities	23	1 (0, 4)	23	0 (0, 3)	0.032^a^
Activity time/day, minutes	21	20 (0, 660)	15	0 (0, 135)	0.695
Activity distance/day, m	19	0 (0, 250)	15	0 (0, 1,000)	0.875
No HCP company					
No. of single activities	23	1 (0, 7)	23	2 (0, 17)	0.185
Activity time/day, minutes	21	30 (0, 420)	15	300 (0, 990)	0.002^a^
Activity distance/day, m	19	0 (0, 300)	15	1,100 (0, 4,000)	0.002^a^

Abbreviation: HCP, health care professional.

The number of available data is reported in the “*n*” column.

a *p*-values that are significant (<0.05) according to the Wilcoxon Signed Rank Test.

## Discussion

Despite the proportion of paracorporeal VADs in long-term mechanical support has increased from 28% to 53% in the pediatric population in the last years,^19^, ^20^ the lack of mobility remains a major obstacle in the management of children on device support.^21^ This study shows improvements in terms of patient activity, mobility, and independence from HCP in children supported with the novel mobile driving unit. Moreover, health related quality of life of the parents caring for them significantly improved as well as measures of depression and stress. In another study, the within-patient comparisons on children with VAD showed a trend toward QoL-improvement after 3 and after 6, respectively, months postimplantation but did not reach statistical significance.^14^ Bed rest and limited physical activity can lead to a sense of lack of control and increase stress levels during the hospitalization period.^22^ Furthermore, prolonged immobility is known to be an important predictor of poor functional outcomes^12^ and exposes patients to significant morbidity, including physical deconditioning, a reduced ability to do activities of daily living, delays in cognitive and social development and mental health issues.^23^, ^24^ In particular, immobilization and prolonged bed rest may be the cause of a wide spectrum of comorbidities, for example, musculoskeletal, cardiovascular, respiratory, endocrine and renal, gastrointestinal, central nervous system, neurodevelopment, balance and coordination complications.^25^, ^26^ On the other hand, early patient mobilization may prevent these complications from occurring and represents the first step in initiating exercise training, which is known to improve the functional capacity and prognosis of HF patients, including those on VAD support.^13^, ^27^, ^28^, ^29^

These aspects play an even more important role when considering the pediatric population. Indeed, in this cohort, a positive association between physical activity and motor and cognitive development, as well as educational benefits, is evidenced.^30^, ^31^, ^32^, ^33^ Mobilization of pediatric patients may also facilitate play, which is another pivotal aspect of childhood development.^34^ Children with chronic or life-threatening illnesses may be limited in their recreative time, which can hinder the achievement of developmental milestones that go beyond the illness itself.^34^ The skills learned in play have a lifelong function and contribute to the development of motor, cognitive, psychosocial, executive function, emotional, and language skills and facilitate resilience creativity and problem-solving skills.^34^, ^35^ Play has a positive impact on children's depression and anxiety rates, and increases the sleep quality.^35^ As such, play is also known to help children cope with the stress of being in hospital for a prolonged period.^22^ Eventually, by enhancing mobility and promoting independence, it is expected that the QoL for patients and their families will be elevated. The ability to engage in activities and experience greater autonomy can have a positive impact on overall well-being and satisfaction with daily life.

The PedsQL results indicate a marked improvement in HRQL of the family on changing from the Ikus to the EXCOR Active, with the greatest differences observed in emotional, social functioning, and daily activity scores (increase in score by 16-20 points). Similarly, the DASS 21 scores demonstrate significant improvements in the levels of depression and stress in patients and their families when switching from the Ikus to the EXCOR Active (drop in score by 5 points). Studies on adults and children with other diseases have shown clinically relevant differences of 4 to 13 for the PedsQL^36^, ^37^ and 4 to 6 for the DASS 21.^38^ However, the comparability of these results with those of our cohort is not supported by evidence. Data from the individual diary revealed improvements in activity time and distance per day and independence from HCP on changing from the Ikus to the EXCOR Active. Taken together, these findings suggest that the EXCOR Active improves mental well-being in patients and caregivers and reduces the negative impact of VAD therapy on the families, in comparison to the Ikus-assisted EXCOR therapy. These aspects are consistent with the known impact of improved mobility on the mental and physical health of VAD patients.^13^, ^27^, ^28^ Several studies in adults have demonstrated that mobilization of these patients, in the form of exercise training, increases various markers of cardiovascular fitness (e.g., functional capacity and muscle strength),^39^, ^40^, ^41^, ^42^ and improves QoL and leads to decrease in depressive symptoms in VAD patients.^41^, ^42^, ^43^ This study extends these findings by showing that, in pediatric patients, the negative impact on the patients’ families is markedly reduced when using a mobile driving unit. An additional benefit of mobility in pediatric patients is its known impact on motor and cognitive development,^30^, ^31^, ^32^, ^33^ which is of particular relevance in children with HF, who are at risk of delays in both cognitive and psychologic functioning.^44^ These findings are in line with this study. The questionnaire results in concert with the diary entries indicate an increase in the overall activity time per day and the total distance covered per day. They also illustrate an increased independence from HCP company. It is tempting to speculate that the increased level of mobility reached by EXCOR Active utilization contributes considerably to the observed augmentation in QoL.

Beyond the findings in QoL, EXCOR Active demonstrated excellent performance and safety profile. The lower incidence of AEs with the novel driving unit is, given the small number of cases, most likely due to chance or could be related to the fact that some AEs such as infections or deposits and neurological events are more frequent in the early postoperative phase.^19^

## Limitations

The study has limitations inherent in all observational studies, that is, being single-arm and nonrandomized, resulting in an inability to prove a causal link between the use of the EXCOR Active driving unit and increases in QoL. In addition, improvements in QoL in the EXCOR Active period may be partially attributable to the normal rehabilitation process after VAD implantation instead of the switch to the mobile driving unit. Also, the self-reported mobility by means of a diary could underlie a subjective bias. However, the observed times spent and distances covered during the activities would not have been possible on the Ikus due to technical restrictions, such as battery life and weight of the device. It should also be noted that the study started during the COVID-19 pandemic. It is acknowledged that this external factor may have affected the stress levels of the patients and their families and their willingness to mobilize. Thus, the pandemic may have had an overall negative impact on the results in the early part of the study.

## Conclusion

This study provides evidence that the novel EXCOR driving unit is both safe and performs well, with excellent outcomes and no safety findings of concern. It also demonstrates that, in comparison to the Ikus, the EXCOR Active driving unit improves mobility in pediatric patients on EXCOR VAD support, which may have significant positive consequences on the ability of the patients to cope with the stress of hospitalization as well as on the long-term neurodevelopmental outcome. Furthermore, the study suggests that the utilization of the EXCOR Active driving unit reduces the negative impact of VAD therapy on patients’ families. The implementation of the EXCOR driving unit could therefore not only benefit the patients but also alleviate the burden and challenges faced by their families.

The results of the study, therefore, provide an important contribution to the management of the cohort of pediatric patients with end-stage HF. The application of this device can be regarded as another step toward outpatient management of children with paracorporeal VADs (Picture 1, Picture 2, Picture 3).

**Picture 1:**
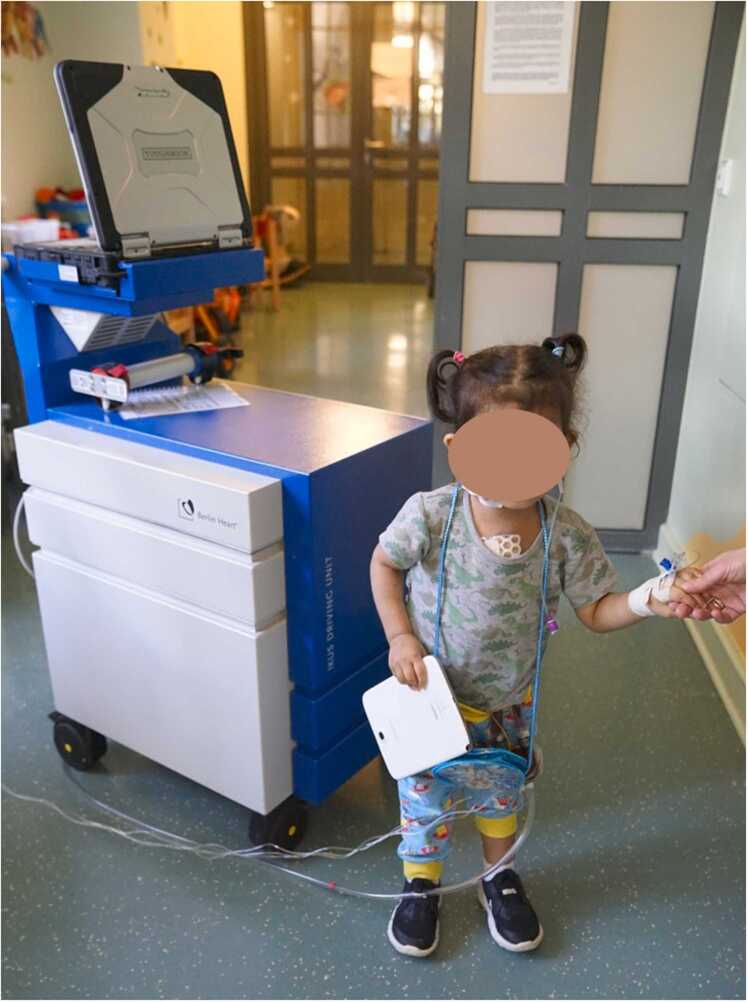
Toddler on Excor VAD support with Ikus driving unit. The driving unit must be pushed by one person with both hands while walking, a second person helps the child and must guide the pneumatic tube.

**Picture 2:**
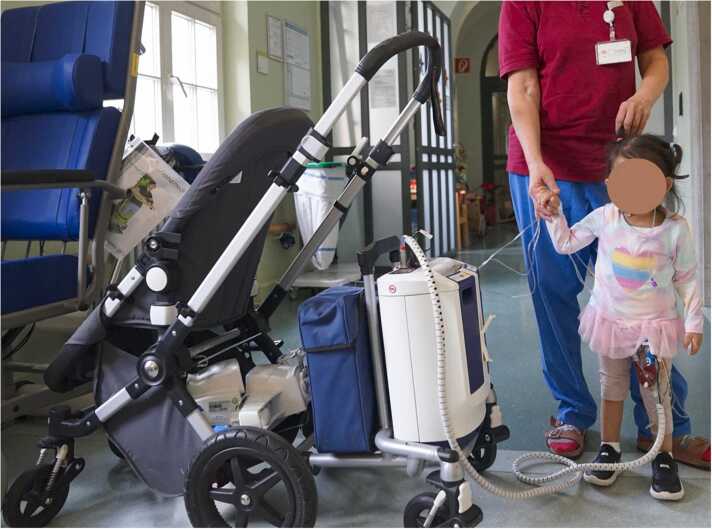
The same child on the Excor Active driving unit, which is attached to a stroller here. To walk, the stroller can be pushed with one hand and the child with the other. The pneumatic tube can be attached to the stroller in a clamp.

**Picture 3:**
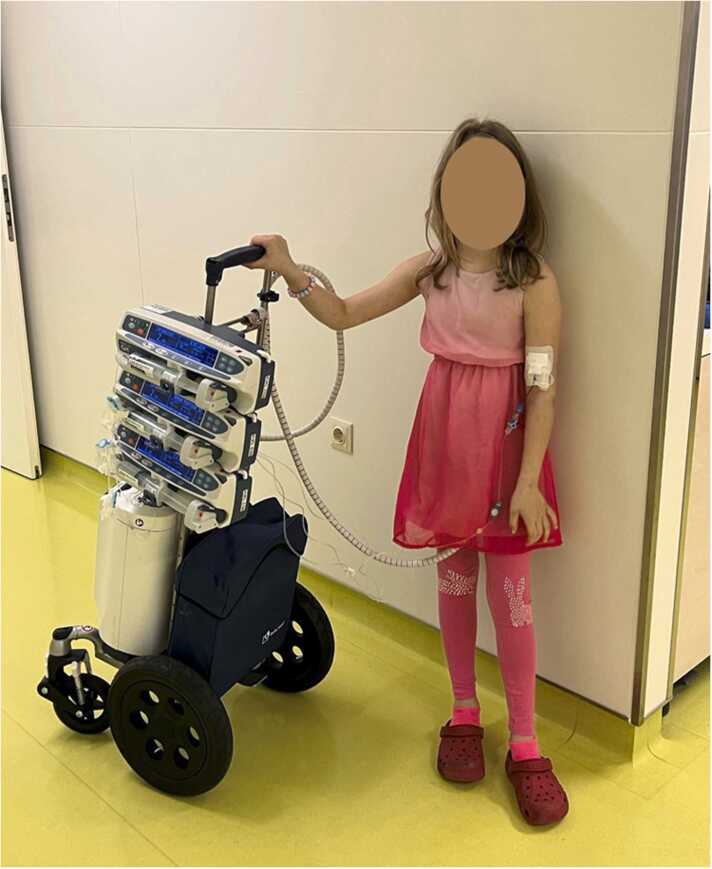
Older children can mobilize on their own.

## Author contributions

O.M. is the coordinating investigator of this clinical study. All authors submitted data and contributed to the data analysis, result interpretation, and manuscript preparation.

## Disclosure statement

The authors declare the following financial interests/personal relationships which may be considered as potential competing interests: O.M. reports a relationship with Berlin Heart that includes travel support and speaking honoraria. E.S. reports a relationship with Berlin Heart that includes travel support, speaking honoraria, and an issued patent. M.S. reports a relationship with Berlin Heart that includes travel support. S.S. reports a relationship with Berlin Heart that includes a research grant. D.Z. reports a relationship with Berlin Heart that includes consulting, travel support, speaking honoraria, and a research grant; with Abbott that includes consulting, travel support, speaking honoraria, and a research grant. All other authors declare no conflict of interest.

The authors wish to thank Berlin Heart, especially Ares Menon, Thomas Schöndorf, Nadia Sinner, and Johanna Aigner for the valuable support in designing and conducting the study, the many fruitful discussions and editorial assistance in preparing the manuscript.

This study was conducted with an unrestricted grant from Berlin Heart.
