# Supplementary material for: Quality of life in pediatric patients on a paracorporeal ventricular assist device with a novel mobile driving system
Source: JHLT Open. 2024 Jul 17;6:100125. doi: 10.1016/j.jhlto.2024.100125 (PMC11935330; doi:10.1016/j.jhlto.2024.100125)
Supplement: Supplementary file 1 — Supplementary material [file mmc1.docx]

Contraindications to being supported by the EXCOR Active (according to IFU):

• Adequate anticoagulation therapy not possible

• Sepsis

• Irreversible multiple organ dysfunction syndrome

• Irreversible respiratory failure

• Anatomical conditions that make implantation surgically impossible

• Aggressive, malignant tumor disease with a very limited life expectancy

• Significantly life-shortening, neurological impairment
