# Supplementary material for: Quality of life in pediatric patients on a paracorporeal ventricular assist device with a novel mobile driving system
Source: JHLT Open. 2024 Jul 17;6:100125. doi: 10.1016/j.jhlto.2024.100125 (PMC11935330; doi:10.1016/j.jhlto.2024.100125)
Supplement: Supplementary file 2 — Supplementary material [file mmc2.docx]

Supplementary diagram: Patients enrolled and cohort providing quality of life questionnaires.
