# Supplementary material for: Quality of life in pediatric patients on a paracorporeal ventricular assist device with a novel mobile driving system
Source: JHLT Open. 2024 Jul 17;6:100125. doi: 10.1016/j.jhlto.2024.100125 (PMC11935330; doi:10.1016/j.jhlto.2024.100125)
Supplement: Supplementary file 3 — Supplementary material [file mmc3.docx]

Supplementary Table 1: Overview of the 7 device deficiencies that occurred during the study observational period. For each device deficiency, it is indicated whether it led to exchange of the driving unit and whether the replacement was assessed as clinically relevant. The reasons for these assessments are given. Device deficiency rates: Ikus: 0.581 events per 100 patient days; 95%CI: 0.015, 3.239; Excor Active: 0.692 events per 100 patient days; 95%CI: 0.254, 1.506. Device unit exchange rates: Ikus 0.58 events/100 patient-days (95% CI: 0.02, 3.24), Excor Active 0.46 events/100 patient-days (95% CI: 0.13, 1.18). N/A, not applicable. In three device exchanges, EXCOR Active was replaced with another EXCOR Active driving unit. In one case EXCOR Active was exchanged with an Ikus (because of limited experience with Excor Active driver, but not for any medical or safety reason), and switched back to EXCOR Active after 2 days.

| **Device** | **Description of the Event** | **Driving Unit Exchanged?** | **Clinically Significant Driving Unit Exchange?** | **Reason for Assessment of Clinical Significance** |
| --- | --- | --- | --- | --- |
| Ikus | Defective battery with many alarms. Driving unit was operating with mains. | Y | N | The driving unit was always fully functional and patient safety was not compromised at any point. |
| EXCOR Active | False positive alarm triggered by conceptual software error. | Y | N | The driving unit was always fully functional and patient safety was not compromised at any point |
| EXCOR Active | Alarm occurred after the driving unit was disconnected from mains and reconnected immediately while the emergency battery was charging. The mains supply was restored before the software recognized it was interrupted. After the mains supply was restored, the charging voltage was not reached again. Thus, the software assumed a hardware defect and emitted an alarm. | Y | N | The driving unit was always fully functional and patient safety was not compromised at any point |
| EXCOR Active | Multiple long-lasting alarms of the flow sensor (flow sensor not connected) due to a loose sensor clip. | Y | N | The driving unit was always fully functional and patient safety was not compromised at any point |
| EXCOR Active | The mean driving pressure could not be set (operator error). In addition, elevated friction levels exceeded the threshold value of 0.07 Ns momentarily, which triggered an alarm. | Y | N | No hemodynamic impairment of patient and patient safety was not compromised at any point |
| EXCOR Active | Three episodes of flow sensor alarms; position of flow sensor was corrected. | N | N/A | N/A |
| EXCOR Active | Battery alarm; battery got exchanged. | N | N/A | N/A |
