# Supplementary material for: Quality of life in pediatric patients on a paracorporeal ventricular assist device with a novel mobile driving system
Source: JHLT Open. 2024 Jul 17;6:100125. doi: 10.1016/j.jhlto.2024.100125 (PMC11935330; doi:10.1016/j.jhlto.2024.100125)
Supplement: Supplementary file 4 — Supplementary material [file mmc4.docx]

Supplementary Table 2: Source data from patient diaries (activity sheets) for visit 2 (Ikus period) (i.e., first diary entry) and at visit 3 (EXCOR® Active period) (i.e., last diary entry).

|  |  | All activities | | HCP company | | No HCP company | |
| --- | --- | --- | --- | --- | --- | --- | --- |
|  |  | Visit 2 | Visit 3 | Visit 2 | Visit 3 | Visit 2 | Visit 3 |
| No. of single activities | Patient 1 | 4 | 6 | 0 | 0 | 2 | 6 |
|  | Patient 21 | 3 | 7 | 0 | 0 | 3 | 7 |
|  | Patient 22 | 8 | 1 | 0 | 0 | 7 | 0 |
|  | Patient 41 | 2 | 1 | 1 | 0 | 1 | 0 |
|  | Patient 61 | 1 | 1 | 1 | 1 | 0 | 0 |
|  | Patient 81 | 2 | 4 | 0 | 0 | 2 | 3 |
|  | Patient 82 | 2 | 3 | 0 | 0 | 2 | 3 |
|  | Patient 101 | 4 | 4 | 1 | 0 | 3 | 4 |
|  | Patient 121 | 5 | 4 | 2 | 1 | 3 | 3 |
|  | Patient 141 | 1 | 1 | 0 | 0 | 1 | 1 |
|  | Patient 161 | 2 | 2 | 2 | 0 | 0 | 2 |
|  | Patient 181 | 3 | 2 | 1 | 1 | 2 | 1 |
|  | Patient 201 | 5 | 18 | 4 | 1 | 1 | 17 |
|  | Patient 221 | 1 | 1 | 0 | 0 | 0 | 0 |
|  | Patient 242 | 2 | 7 | 1 | 3 | 0 | 4 |
|  | Patient 261 | 2 | 2 | 2 | 0 | 0 | 2 |
|  | Patient 281 | 1 | 1 | 0 | 0 | 0 | 1 |
|  | Patient 301 | 4 | 1 | 1 | 0 | 2 | 0 |
|  | Patient 321 | 1 | 2 | 1 | 0 | 0 | 2 |
|  | Patient 341 | 4 | 3 | 0 | 1 | 4 | 2 |
|  | Patient 361 | 2 | 1 | 2 | 0 | 0 | 0 |
|  | Patient 381 | 1 | 1 | 1 | 0 | 0 | 0 |
|  | Patient 401 | 2 | 1 | 1 | 0 | 1 | 0 |
| Activity time/day, min | Patient 1 | 265 | 300 | 0 | 0 | 180 | 300 |
|  | Patient 21 | 80 | 310 | 0 | 0 | 80 | 310 |
|  | Patient 22 | 420 | . | 0 | . | 420 | . |
|  | Patient 41 | 100 | . | 30 | . | 70 | . |
|  | Patient 61 | 20 | 120 | 20 | 120 | 0 | 0 |
|  | Patient 81 | 80 | 570 | 0 | 0 | 80 | 360 |
|  | Patient 82 | 132 | 990 | 0 | 0 | 132 | 990 |
|  | Patient 101 | 285 | 240 | 25 | 0 | 260 | 240 |
|  | Patient 121 | 150 | 465 | 50 | 30 | 100 | 435 |
|  | Patient 141 | 20 | 240 | 0 | 0 | 20 | 240 |
|  | Patient 161 | 660 | 360 | 660 | 0 | 0 | 360 |
|  | Patient 181 | 45 | 45 | 15 | 30 | 30 | 15 |
|  | Patient 201 | 131 | 745 | 101 | 40 | 30 | 705 |
|  | Patient 221 | . | . | . | . | . | . |
|  | Patient 242 | 15 | 450 | 15 | 135 | 0 | 315 |
|  | Patient 261 | 50 | 105 | 50 | 0 | 0 | 105 |
|  | Patient 281 | . | . | . | . | . | . |
|  | Patient 301 | 158 | . | 11 | . | 120 | . |
|  | Patient 321 | 30 | 120 | 30 | 0 | 0 | 120 |
|  | Patient 341 | 170 | 165 | 0 | 45 | 170 | 120 |
|  | Patient 361 | 70 | . | 70 | . | 0 | . |
|  | Patient 381 | 135 | . | 135 | . | 0 | . |
|  | Patient 401 | 45 | . | 25 | . | 20 | . |
| Activity distance/day, m | Patient 1 | 450,00 | 4000,00 | ,00 | ,00 | 300,00 | 4000,00 |
|  | Patient 21 | 300,00 | 1475,00 | ,00 | ,00 | 300,00 | 1475,00 |
|  | Patient 22 | 107,73 | . | ,00 | . | 105,84 | . |
|  | Patient 41 | 15,00 | . | 15,00 | . | ,00 | . |
|  | Patient 61 | 30,00 | 1000,00 | 30,00 | 1000,00 | ,00 | 1000,00 |
|  | Patient 81 | 252,00 | 3800,00 | ,00 | ,00 | 252,00 | 3800,00 |
|  | Patient 82 | 220,00 | 2240,00 | ,00 | ,00 | 220,00 | 2240,00 |
|  | Patient 101 | 250,00 | 1110,00 | 250,00 | ,00 | ,00 | 1110,00 |
|  | Patient 121 | . | 2100,00 | . | ,00 | . | 2100,00 |
|  | Patient 141 | 15,00 | 800,00 | ,00 | ,00 | 15,00 | 800,00 |
|  | Patient 161 | ,00 | 3276,92 | ,00 | ,00 | ,00 | 3276,92 |
|  | Patient 181 | ,00 | ,00 | ,00 | ,00 | ,00 | ,00 |
|  | Patient 201 | 165,00 | 108,00 | 165,00 | ,00 | ,00 | 108,00 |
|  | Patient 221 | . | . | . | . | . | . |
|  | Patient 242 | ,00 | 180,00 | ,00 | ,00 | ,00 | 180,00 |
|  | Patient 261 | . | 1500,00 | . | ,00 | . | 1500,00 |
|  | Patient 281 | . | 500,00 | . | ,00 | . | 500,00 |
|  | Patient 301 | 120,00 | . | 60,00 | . | 60,00 | . |
|  | Patient 321 | 20,00 | 550,00 | 20,00 | ,00 | ,00 | 550,00 |
|  | Patient 341 | ,00 | . | ,00 | . | ,00 | . |
|  | Patient 361 | ,00 | . | ,00 | . | ,00 | . |
|  | Patient 381 | ,00 | . | ,00 | . | ,00 | . |
|  | Patient 401 | 150,00 | . | 100,00 | . | 50,00 | . |

Abbreviations: HCP, healthcare professional.
